# Recent advances in overcoming barriers to cell‐based delivery systems for cancer immunotherapy

**DOI:** 10.1002/EXP.20210106

**Published:** 2022-03-15

**Authors:** Yingyue Ding, Yixin Wang, Quanyin Hu

**Affiliations:** ^1^ Pharmaceutical Sciences Division School of Pharmacy University of Wisconsin–Madison Madison Wisconsin USA; ^2^ Carbone Cancer Center School of Medicine and Public Health University of Wisconsin–Madison Madison Wisconsin USA; ^3^ Wisconsin Center for NanoBioSystems School of Pharmacy University of Wisconsin–Madison Madison Wisconsin USA

**Keywords:** cancer, cell, delivery barriers, drug delivery, immunotherapy

## Abstract

Immunotherapy strategies that use cell‐based delivery systems have sparked much interest in the treatment of malignancies, owing to their high biocompatibility, excellent tumor targeting capability, and unique biofunctionalities in the tumor growth process. A variety of design principles for cell‐based immunotherapy, including cell surface decoration, cell membrane coating, cell encapsulation, genetically engineered cell, and cell‐derived exosomes, give cancer immunotherapy great potential to improve therapeutic efficacy and reduce adverse effects. However, the treatment efficacy of cell‐based delivery methods for immunotherapy is still limited, and practical uses are hampered due to complex physiological and immunological obstacles, such as physical barriers to immune infiltration, immunosuppressive tumor microenvironment, upregulation of immunosuppressive pathways, and metabolic restriction. In this review, we present an overview of the design principles of cell‐based delivery systems in cancer immunotherapy to maximize the therapeutic impact, along with anatomical, metabolic, and immunological impediments in using cell‐based immunotherapy to treat cancer. Following that, a summary of novel delivery strategies that have been created to overcome these obstacles to cell‐based immunotherapeutic delivery systems is provided. Also, the obstacles and prospects of next‐step development of cell‐based delivery systems for cancer immunotherapy are concluded in the end.

## Graphical abstract

This review covers the design of cell‐based delivery systems for cancer immunotherapy, as well as the significant challenges limiting the efficacy of cell‐based immunotherapy and summarizes recent advances in overcoming those barriers to improve cancer treatment outcomes.

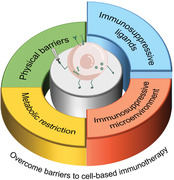

## 1 INTRODUCTION

As understanding of the critical role of the human body's immune system against cancer has advanced, tumor immunotherapy has attracted tremendous enthusiasm in favor of improved overall survival rate and long‐term therapeutic benefits without significant side effects. Various immunotherapeutic approaches emerged as promising anti‐tumor modalities by regulating/promoting the patient's own immune system to kill cancer cells, including immunostimulatory cytokines‐mediated therapy, immune checkpoint blockade, monoclonal antibody‐dependent cellular cytotoxicity treatment, and adoptive immune cell transfer.^[^ ^1^ ^]^ Especially, unleashing anti‐cancer immunity with immune checkpoint inhibitors, including inhibitors blocking cytotoxic T‐lymphocyte‐associated protein‐4 (CTLA‐4), programmed death protein‐1 (PD‐1), and programmed death‐ligand 1 (PD‐L1), and the adoptive transfer of chimeric antigen receptors T cells (CAR‐T cells), has been widely indicated in clinical applications against a variety of cancer.^[^ ^2^, ^3^ ^]^ As of Dec. 2020, six anti‐PD‐1 or anti‐PD‐L1 antibodies have been approved by the United States Food and Drug Administration (FDA) for the treatment of a broad array of cancer.^[^ ^4^ ^]^ Besides, CAR‐T therapy has achieved great success in combating hematological malignancies and solid tumors in clinical trials.^[^ ^5^ ^]^ Notably, combinational immunotherapy approaches by integrating multiple therapeutic modalities or same treatment modality for different immune targets display excellent synergistic anti‐tumor effects, resulting in enhanced treatment outcomes and diminished harmful side effects.^[^ ^6^ ^]^ Despite the great success achieved by cancer immunotherapy for clinical translation, there are still multiple factors hampering the further application of immunotherapy, such as variable objective response rate across patients, normal tissue damage due to off‐target effects, cytokines storm in adoptive CAR‐T cell therapy.

Innovative and effective drug delivery technology has been widely explored in recent years, with a more profound knowledge of biocompatible materials and cancer immuno‐oncology, in order to enhance the efficacy of cancer immunotherapy. Delivery systems that have better accessibility to tumor sites or immune organs, reduced off‐target adverse effects, cargo protection ability, and bio‐responsive and controlled release of cargoes have been applied to facilitate cancer immunotherapy for better both efficacy and safety.^[^ ^7^, ^8^ ^]^ Nanoparticles, biomaterial implant scaffolds, and transdermal delivery systems are being widely investigated as more efficient delivery platforms in cancer immunotherapy for better therapeutic outcome.^[^ ^8^ ^]^ Significantly, the emergence and rapid development of nanotechnology accelerate the research progress of the application of nanoparticle‐based delivery systems in cancer diagnosis and treatment, by reason of enhanced permeability and retention effect and active targeting capability, and also the ability to help to overcome drug resistance.^[^ ^9^, ^10^ ^]^ Despite these remarkable properties of nanotechnology‐enabled drug delivery systems, the drawbacks of nanomaterials, such as immunogenicity, rapid clearance, and safety issues for long‐term administration, raise concerns about their clinical translation.^[^ ^11^ ^]^ Unlike nano‐based delivery systems, cell‐inspired delivery platforms have been raising great attraction owing to their outstanding biocompatibility and the distinctive biological functions of different cell types, including long‐term circulation, low immunogenicity, and low cytotoxicity.^[^ ^12^ ^]^ For example, immune cell‐based delivery systems could actively target and penetrate deeply into the tumor parenchyma responding to inflammatory and hypoxia tumor microenvironment.^[^ ^13^ ^]^ Meanwhile, erythrocyte‐based delivery systems have demonstrated the great biocompatibility and long circulation time.^[^ ^14^ ^]^ As a result, cell‐based delivery methods hold a lot of potential for enhancing the efficacy and safety of cancer immunotherapeutics.

Efficient anti‐tumor immunity enhanced by cancer immunotherapies delivered via cell‐based delivery platforms necessitate a highly coordinated, powerful, and timely anti‐tumor immune response based on the ability to control or eliminate cancer cells via diverse immunity pathways. Briefly, in order to destroy cancer cells and generate long‐term immunologic memory, immune effector cells must migrate towards tumor tissues through complex tumor‐associated blood vasculature and penetrate tumor tissues effectively, then recognize tumor‐associated antigens presented by antigen presenting cells such as dendritic cells.^[^ ^15^ ^]^ However, to escape from the immune surveillance and attack, cancer cells have developed mechanisms to elude immune cell recognition and eradication by regulating molecular expression,^[^ ^16^ ^]^ restraining the activity of immune effector cells,^[^ ^17^ ^]^ and creating the tumor growth‐favorable and immunosuppressive microenvironment,^[^ ^18^ ^]^ which collectively limit the therapeutic efficacy of cancer immunotherapy and become general barriers to eliciting robust anti‐tumor immune response.^[^ ^19^, ^20^, ^21^ ^]^ These inherent obstacles, especially the hindered immune infiltration due to physical barriers, the overexpression of immunoinhibitory ligands on tumor cells, the recruitment of immunosuppressive cells in the tumor microenvironment, and metabolic restriction, largely limit the treatment outcome of immunotherapy that has been facilitated by cell‐based delivery systems.^[^ ^22^ ^]^ Therefore, overcoming these innate barriers to further enhance the therapeutic efficacy of cell‐carried immunotherapeutics has become a central theme in cancer immunotherapy area.

**FIGURE 1:**
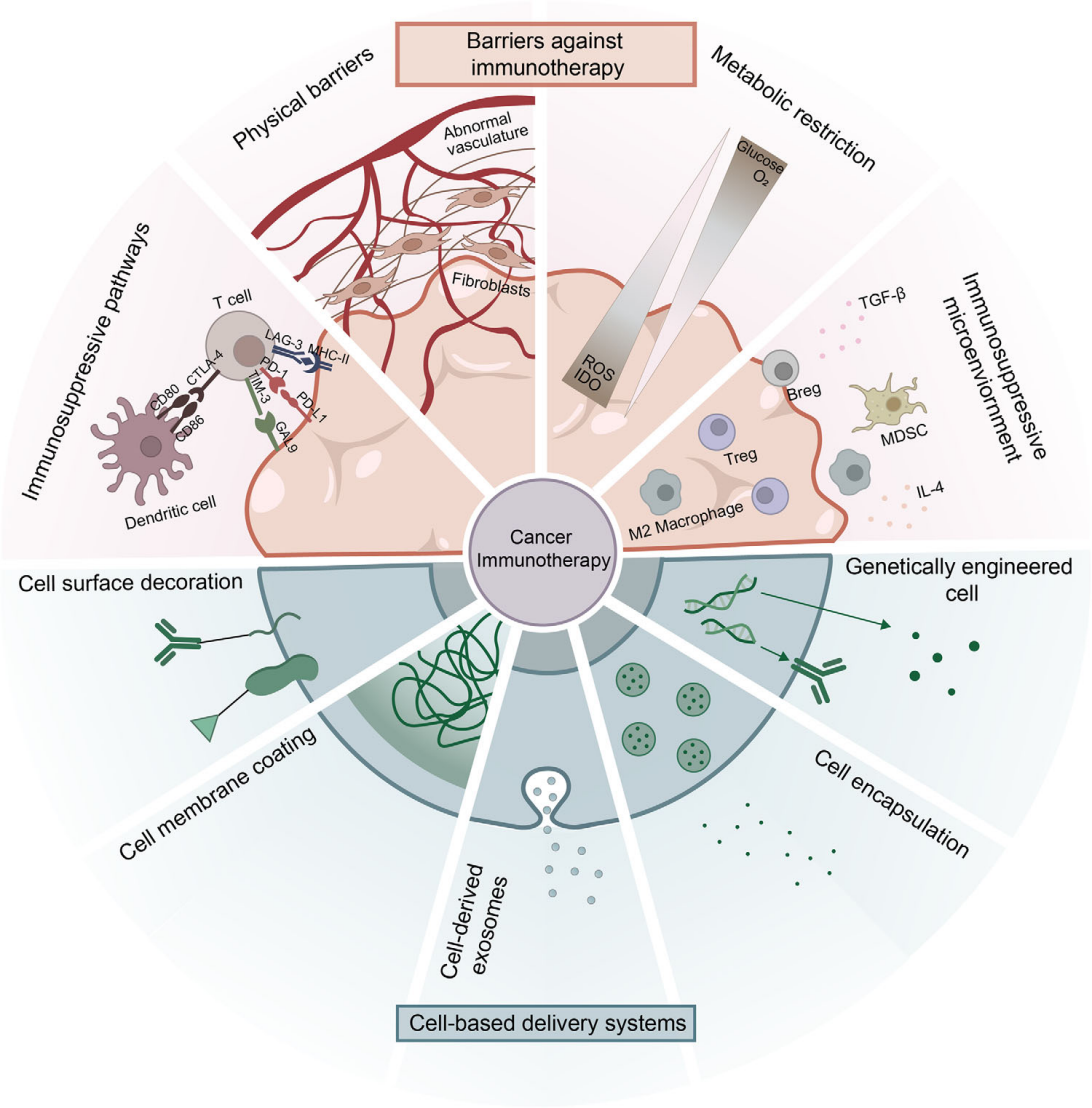
Schematic illustration of design principles of cell‐based delivery systems in cancer immunotherapy and therapeutical barriers to cancer immunotherapy

In this review, we summarize the most widely implemented cell types as carriers of immunotherapeutic agents and the rationale of designing cell‐based delivery strategies for cancer immunotherapy, focusing on cell surface decoration/conjugation, cell membrane coating, cell‐derived exosomes, cell encapsulation, and cell genetic engineering, with specific biomedical applications (Figure 1). Furthermore, the physiological, immunological, and metabolic constraints of cell‐based delivery methods in cancer immunotherapy are discussed, as well as current advances in developing immunotherapeutic techniques to address these limitations. Finally, the future of cell‐based delivery systems in cancer immunotherapy is outlooked with the emphasis on the multidisciplinary collaboration to overcome fundamental limitations against future clinical translation.

## 2 DESIGN PRINCIPLES OF CELL‐BASED DELIVERY SYSTEMS IN CANCER IMMUNOTHERAPY

As the most important units in human bodies, diverse types of cells with different physiological activities are serving as natural biological drug delivery systems in cancer immunotherapy. Erythrocytes, immune cells, stem cells, platelets, neutrophils, cancer cells, and bacteria have been studied as drug delivery carriers, taking advantage of their unique biological or structural functions in transportation, interaction, and immune activity.^[^ ^23^ ^]^ For instance, red blood cells with long circulation life in the bloodstream play an essential role in improving the half‐life time of therapeutic agents and also protect cargos from rapid in vivo degradation and clearance.^[^ ^24^, ^25^ ^]^ Moreover, inspired by the roles of immune cells in the anti‐tumor immune response, drug delivery vehicles based on autologous immune‐related cells show great promise in active targeting and responsiveness to inflammation and infection.^[^ ^26^ ^]^ CAR‐T cell therapy is an excellent example of employing in vitro engineered T cells expressing chimeric antigen receptors to target specific tumor antigens, further leading to T cell proliferation and cytotoxicity against tumor cells. Because of the promising achievement in treating lymphoma and myeloma in clinical trials, two FDA‐approved CAR‐T cell therapies targeting B lymphocyte antigens, lisocabtagene maraleucel and idecabtagene vicleucel, have been commercialized.^[^ ^27^, ^28^ ^]^ Biological, chemical, and genetic engineering approaches have been widely used on whole cells or cell components like cell membrane to develop effective therapeutic carriers in cancer immunotherapy to exploit the inherent characteristics of cells fully. Five types of design concepts for cell‐based delivery systems in cancer immunotherapy have been widely studied so far: cell surface decoration/conjugation, cell membrane coating, cell‐derived exosomes, cell encapsulation, and genetically engineered cell (Table 1).

**TABLE 1:** Summary of the pros and cons of the current design principles of cell‐based delivery systems in cancer immunotherapy

**Design principles of cell‐based delivery systems in cancer immunotherapy**	**Pros**	**Cons**
Cell surface decoration/conjugation	(i) Various functional groups available for chemical conjugation and biorthogonal binding of immunotherapeutic agents (ii) Flexibility for a wide range of decorating moieties with different properties	(i) Cytotoxicity to carrier cells (ii) Impaired circulation time due to interaction with surrounding biologicals after surface conjugation
Cell membrane coating	(i) Prolonging the circulation time of delivery carrier and mitigating immune clearance (ii) Maintaining partial biological characteristics of source cells	(i) Scale‐up production problems (ii) The restricted source of cells for membrane extraction
Cell‐derived exosomes	(i) High biocompatibility, extended circulation half‐life time (ii) Preserving immunomodulation functions inherited from source cells (iii) Off‐the‐Shelf capability	(i) Scale‐up production due to the limited yield (ii) Relatively low circulation stability
Cells as encapsulation carriers	(i) Long‐term release and better biodistribution of loaded immunotherapeutic drugs (ii) Protection of the fragile therapeutics	The possibility of the unstable drug preservation inside cells due to the complex intracellular physiological environment
Genetically engineered cells	Flexibly endowing carrier cells with specific functional features as designed	(i) Permanent change in the genome of source cells (ii) Off‐target risks

### 2.1 Cell surface decoration/conjugation

Because of its established manufacturing process and wide range of modification options, cell surface engineering has been prioritized in developing cell‐based delivery systems. Proteins, lipids, and carbohydrates are diverse components of cell membranes exposing functional groups such as amine and thiol groups in lysine and cysteine residues, which are ideal for chemically conjugating immunotherapeutic agents on the surface of cell carriers through facile chemical reactions.^[^ ^29^, ^30^ ^]^ *N*‐hydroxyl‐succinimidyl ester groups, maleimide, and 1‐ethyl‐3‐(3‐dimethyl aminopropyl) carbodiimide are widely used as chemical cross‐linkers for chemical conjugation of biological or chemical materials on the cell surface.^[^ ^13^, ^31^ ^]^ Also, biorthogonal binding sites could be formed on cell membranes by metabolically integrating non‐natural sugars in order to create stable covalent conjugates with therapeutic materials, thereby reducing the negative impact of cell surface modification on normal biological processes.^[^ ^32^ ^]^ In addition to covalent conjugation methods based on the diversity of functional moieties on the cell membrane, other cell surface engineering approaches are also gaining attention, such as hydrophobic insertion of amphiphilic materials with hydrophobic chains like polyethylene glycol into lipid bilayers and electrostatic self‐assembly between the anionic cell membranes and cationic polymers.^[^ ^33^ ^]^ As a promising approach to develop cell‐based delivery systems, cell surface decorating strategy has been used to investigate the prospect of a wide range of cells serving as drug delivery vehicles, including red blood cells,^[^ ^34^ ^]^ platelets,^[^ ^35^ ^]^ dendritic cells,^[^ ^36^ ^]^ macrophages,^[^ ^37^ ^]^ T cells,^[^ ^38^ ^]^ and even bacteria.^[^ ^39^ ^]^ Although cell surface engineering has been shown to be a safe and reliable approach for creating cell‐based delivery systems in cancer immunotherapy, concerns about cytotoxicity to carrier cells and a lack of stability in systemic circulation persist.

### 2.2 Cell membrane coating

Cell membranes generated from natural cells have shown to be an excellent choice for camouflaging synthetic nanoparticles for better drug delivery in cancer immunotherapy. Usually, due to the concern about the biocompatibility of foreign nanoparticles of which capture and clearance occurred rapidly by reticuloendothelial system, strategies that could prolong the circulation time of nanoparticles, help nanoparticles escape from immune clearance, and lead to specific tropism to tumor sites, are pursued by scientists actively.^[^ ^40^ ^]^ By coating nanoparticles with cell membrane‐derived vesicles that partially maintain their original biological characteristics like the expression of specific membrane proteins and unique moieties, the enhanced pharmacokinetic profile and tumor targeting ability of immunotherapeutic‐loaded nanoparticles have been observed with the improved anti‐tumor efficacy.^[^ ^41^ ^]^ So far, red blood cell membrane,^[^ ^42^ ^]^ platelet membrane,^[^ ^43^ ^]^ macrophage membrane,^[^ ^44^ ^]^ dendritic cell membrane,^[^ ^45^ ^]^ natural killer cell membrane,^[^ ^46^ ^]^ and cancer cell membrane^[^ ^47^ ^]^ have been studied for cell membrane wrapping, demonstrating their unique advantages of drug delivery in cancer immunotherapy. Besides a wide range of options in cell membrane sources, nanoparticles could be successfully coated with great flexibility in their hydrophobicity, size, or structure, providing great potential for the application of cell membrane coating in various research fields like imaging, vaccination, and drug delivery.^[^ ^13^ ^]^

The inherent characteristics of different original cells are preserved by different types of cell membranes extracted by top‐down and microfluidic electroporation methods.^[^ ^48^ ^]^ Tumor cell membrane‐coated nanoparticles, for example, demonstrated an excellent specific targeting capacity towards homologous tumor cells. Furthermore, tumor cell membrane proteins might be used as antigens in cancer vaccines to enhance anti‐tumor immunity and provide long‐term immune response.^[^ ^49^ ^]^ While for leukocyte membrane‐coated nanoparticles, preferential accumulation for inflammation sites is the most crucial benefit for effectively targeting tumor inflammatory microenvironment.^[^ ^50^ ^]^ Besides, hybrid membrane‐coated nanoparticles are produced by fusing two separate cell membranes to wrap nanoparticles in order to combine or enhance the benefits of different types of cell membranes. For instance, red blood cell and cancer cell‐fused membranes have been used to coat nanoparticles as effective delivery vehicles for cancer vaccines and immune checkpoint inhibitors, with cancer cell membrane serving as the component that provides tumor associated antigens and red blood cell membrane having the inherent ability to be captured by antigen presenting cells.^[^ ^42^ ^]^ Despite the fact that cell membrane‐coated nanoparticles have made significant progress in improving immunotherapy, clinical translation remains a challenge. Scale‐up production necessitates consistent quality control requirements, which include even and complete covering of cell membranes on nanoparticles with the right orientation, as well as the preservation of functional surface proteins on the cell membranes.^[^ ^40^ ^]^ Furthermore, due to the challenges of large‐scale ex vivo culturing and the short lifetimes of certain types of cells, the restricted source of cells for membrane extraction barely meets the need of the considerable amount of cell membrane‐coated nanoparticles needed for effective immunotherapy in the clinic.

### 2.3 Cell‐derived exosomes

Due to their immunogenicity and signaling transfer capabilities, cell‐derived exosomes, as naturally generated nano‐sized extracellular vesicles, play a role in the regulation of immune responses.^[^ ^51^ ^]^ In the context of cancer, exosomes produced from tumor cells and immune‐related cells can regulate tumor antigen presentation, cause immunological activation and suppression, and engage in immune surveillance, all of which have sparked interest in using exosomes as immune modulators in immunotherapy to activate immune systems against tumor development and metastasis.^[^ ^52^ ^]^ Besides the immunomodulation function, exosomes as drug delivery carriers in cancer immunotherapy have several additional benefits, including the capacity to transport mRNA, lipids, and proteins, as well as high biocompatibility and extended circulation duration.^[^ ^53^ ^]^ Tumor cells,^[^ ^54^ ^]^ dendritic cells,^[^ ^55^ ^]^ T cells,^[^ ^56^ ^]^ natural killer cells,^[^ ^57^ ^]^ platelets,^[^ ^58^ ^]^ bacteria,^[^ ^59^ ^]^ and macrophages^[^ ^60^ ^]^ have all been described as feasible cell sources for producing carrier exosomes with biological functions. Exosomes produced from natural killer cells, for example, contain molecular markers such as lineage marker CD56 and activating receptor natural killer group 2D, causing tumor cells to undergo substantial apoptosis.^[^ ^61^ ^]^ Furthermore, activated CD8+ T cell extracellular vesicles could help to inhibit tumor invasion and metastasis by acquiring mesenchymal‐like characteristics.^[^ ^62^ ^]^ As a result of the advantages listed above, cell‐derived exosomes are very promising to be developed for therapeutic use for cancer immunotherapy. Passive and active cargo‐loading procedures are the two main engineering approaches for loading therapeutic substances into exosomes.^[^ ^53^ ^]^ In a brief, therapeutic medicines may be loaded by simply co‐incubating with source cells or cell‐derived exosomes without any further chemical or physical treatments. And to further promote encapsulation effectiveness, passive loading techniques like sonication, electroporation, and incubation with membrane permeabilizers are also applied.^[^ ^53^ ^]^ Taking sonication as an example, when exosomes are mixed with drug molecules and then sonicated with a homogenizer probe, the probe‐generated mechanical shear stress destabilizes the exosomes' membrane, allowing the drug to infiltrate into the exosomes. Despite the fact that a variety of engineering approaches for developing exosome‐based immunotherapy are accessible, there are still some challenges with limited exosome yield, exosome extraction, and large‐scale manufacturing, which restrict their clinical translation.

### 2.4 Cells as encapsulation carriers

Encapsulating immunotherapeutic drugs in the intracellular milieu of cell carriers is a promising delivery approach, as it makes use of the inner space of cells as reservoirs for long‐term release and better biodistribution. Loading cargos into inner compartment of the cell require different engineering approaches like electroporation,^[^ ^63^ ^]^ osmosis‐based methods,^[^ ^64^ ^]^ and co‐incubation,^[^ ^65^ ^]^ according to the particular features of specific cell types. Incubating therapeutic compounds with circulating cells that exhibit the features of natural phagocytosis mechanisms, such as neutrophils and macrophages, is the simplest way of cargo internalization ex vivo.^[^ ^66^ ^]^ This method was widely utilized in the nanoparticle loading process due to the unique endocytosis features of nano‐sized fragments by cells.^[^ ^67^ ^]^ More intriguingly, anti‐tumor hydrophobic drugs could also be encapsulated by adipocytes through the lipid droplet uptake mechanisms. Wen et al. developed adipocytes as drug delivery depots that could achieve tumor‐site local and sustained release of anti‐cancer rumenic acid and reactive oxygen species (ROS)‐responsive doxorubicin prodrug, resulting in downregulation of PD‐L1 expression and T cell‐mediated anti‐tumor immunity.^[^ ^68^ ^]^ In addition to utilizing the nature of cells to encapsulate drugs, external stimulation that changes the physiological structure of the cell membrane to generate drug‐passable holes is also an essential technique of loading immunotherapeutic drugs. Several studies have proved the feasibility of using hypotonic dialysis to enhance the permeability of red blood cell membranes to transport drugs or nanoparticles into cell carriers based on osmotic pressure difference.^[^ ^69^, ^70^ ^]^ When the osmotic pressure returns to normal, the structure of drug‐loaded red blood cells will restore. Other methods like electroporation could also trigger the drug encapsulation by pore‐forming on the platelet or red blood cell membrane,^[^ ^71^ ^]^ but concern about whether electroporation would influence the standard functionality of carrier cells still remains. Despite the fact that the anti‐tumor efficacy and tumor targeting ability of the treatment is largely advanced, the possibility of unstable drug preservation inside cells due to complex intracellular physiological environment with lysosome clearance and enzyme degradation,^[^ ^72^ ^]^ further causing uncontrolled drug leakage and undesired side effects on the carrier cells or non‐targeted tissues in the body.^[^ ^13^ ^]^

### 2.5 Genetically engineered cell

Recently, the phenotype and functional alteration of carrier cells for drug delivery achieved by genetic engineering is booming because of the promising development of viral and non‐viral vehicle‐based gene‐editing tools.^[^ ^73^ ^]^ By endowing carrier cells with dynamic features, including the upregulation of TNF‐related apoptosis‐inducing ligand expression,^[^ ^74^ ^]^ enhanced secretions of pro‐inflammatory cytokines to boost anti‐cancer immunity,^[^ ^75^ ^]^ and targeting specificity toward tumor surface antigens,^[^ ^76^ ^]^ genetically engineered cells have achieved significant success with robust and long‐term anti‐tumor functions, eliminating the tumor growth and metastasis. CAR‐T cell therapy, one of the most promising cancer immunotherapeutic strategies, is a great example in which ex vivo engineered T cells are modified to express chimeric antigen receptors by lentiviral or σ‐retroviral‐based transduction methods to target tumors and generate anti‐tumor effector T cells.^[^ ^77^, ^78^ ^]^ The production of chimeric antigen receptors could also be carefully regulated by particular circuits to locally stimulate CAR expression, which is known as synthetic Notch (SynNotch) CAR‐T treatment, to maximize the specificity of tumor cell identification and avoid side effects against healthy tissues.^[^ ^79^ ^]^ Choe et al. developed a SynNotch CAR‐T treatment that could recognize multi‐antigen combinations and largely improved the specificity of CAR‐T treatment.^[^ ^80^ ^]^ SynNotch receptors were primed with EGFRvIII neoantigen, which is glioblastoma‐specific but heterogeneous, to trigger the expression of downstream homogeneous EphA2 and IL13Rα2 antigens for glioblastoma, which showed an unsatisfactory therapeutic outcome as a single CAR due to the unwanted systemic toxicity. Therefore, this prime‐and‐kill circuit, by combining two CARs, could largely enhance the glioblastoma cell targeting specificity and avoid the off‐target toxicity. Aside from direct ex vivo gene modification of cell carriers, editing genes of stem cells as precursors to obtain specific gene‐expressed differentiated cells is another approach for generating genetically engineered cell carriers, which is suitable for modifying nonnucleated cells like platelets.^[^ ^81^ ^]^ However, without thorough studies, it remains elusive whether differentiated cells would stably express edited genes as predicted and whether differentiated functional cells will maintain good biofunctionalities after multiple generations.

## 3 BARRIERS TO EFFECTIVE CANCER IMMUNOTHERAPY DELIVERED BY CELL‐BASED CARRIERS

### 3.1 Physical barriers to immune infiltration

Physical barriers erected during tumor progression and metastasis, including the dysregulated extracellular matrix (ECM) containing excessive fibrillar collagen and hyaluronic acid (HA), tumor‐associated fibroblasts in the tumor microenvironment, and abnormal tumor vasculature, have shown to be critical to overcome for effective cancer immunotherapy delivered by cell‐based drug carriers. As one of the cancerous physiological characteristics, ECM undergoes structural change because of the tumor‐associated fibroblasts in the tumor microenvironment, which are capable of producing structural macromolecules, including collagen, fibronectin, and HA, resulting in the increased matrix stiffness and high interstitial pressure in ECM.^[^ ^82^ ^]^ Studies have shown that a high density of type I collagen can promote the malignant transformation of epithelial cells and create stiff ECM, preventing CD8+ T lymphocytes infiltration and activation at tumor sites.^[^ ^83^ ^]^ In addition, HA has been proved to be correlated to the recruitment and activation of stromal cells, tumor angiogenesis, and lymphangiogenesis, which leads to more aggressive malignancy.^[^ ^84^, ^85^, ^86^ ^]^ Besides establishing thick ECM barriers to restrain the immune cell infiltration, stiff ECM would also enhance the motility and invasion of tumor cells through multiple signaling pathways.^[^ ^82^, ^87^ ^]^ Consequently, although autologous T cells employed as carriers to deliver drug‐loaded nanoparticles have demonstrated remarkable targeting capacity to treat particular types of malignancies like Burkitt's lymphoma compared to free nanoparticles,^[^ ^88^ ^]^ T cell‐mediated anti‐tumor immunity was still largely limited due to the immunosuppressive property of dense ECM, which inhibited immune cell infiltration, resulting in the unsatisfactory therapeutic efficacy of cancer immunotherapy, particularly immunotherapeutics delivered by immune cells like CAR‐T cells. To maximize the therapeutic effect of cancer immunotherapy with cell‐based delivery methods, targeting ECM components and tumor‐associated fibroblasts to cooperate with immunotherapy becomes a promising strategy and attracts rising attention.

**FIGURE 2:**
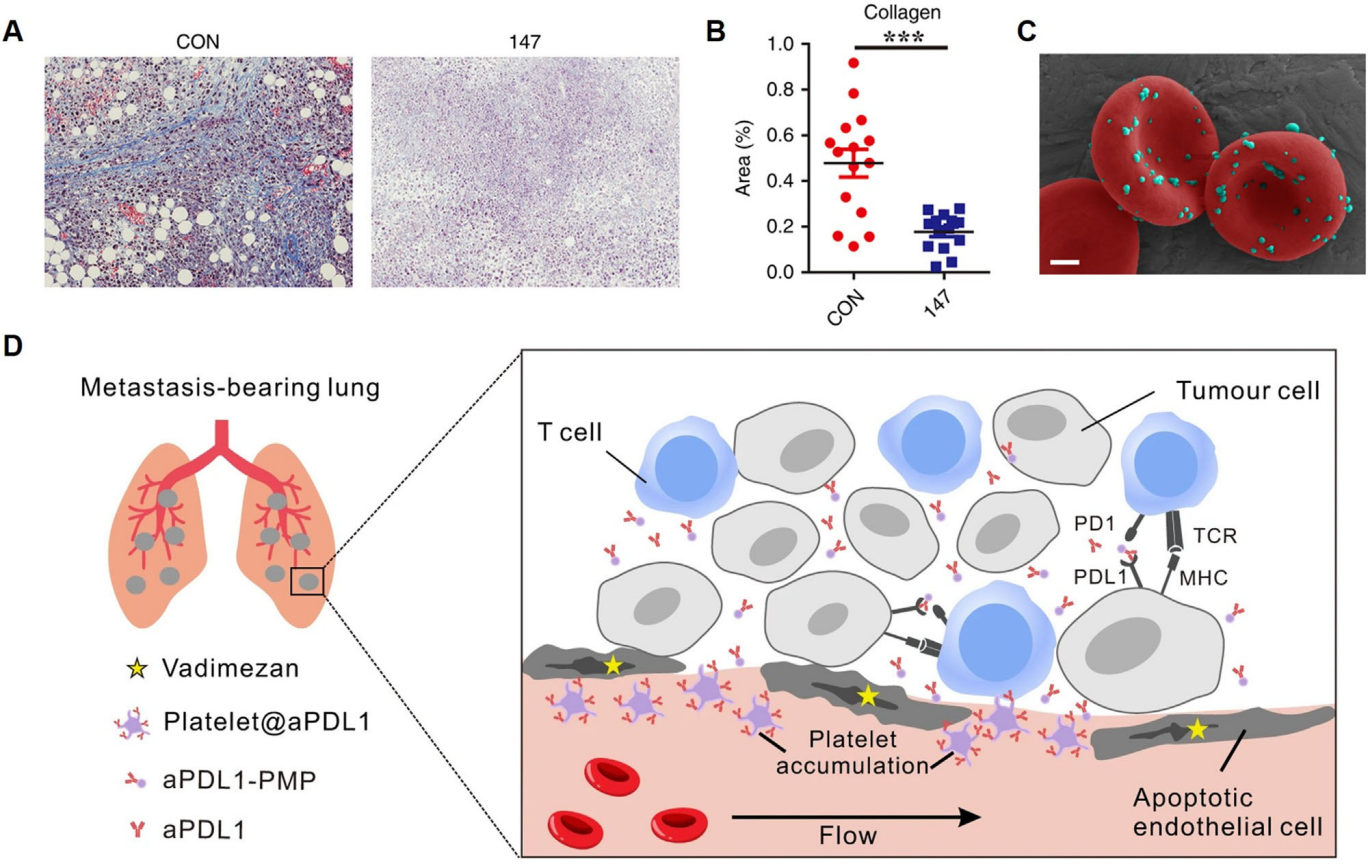
Overcoming physical barriers to immune infiltration. (A) Representative images of tumor sections showing stained extracellular matrix deposition after treating with CAR‐147 macrophages. (B) Quantified analysis of the extracellular matrix. Reproduced with permission.^[^ ^94^ ^]^ Copyright 2019, The Author(s), under exclusive license to Cancer Research UK. (C) Scanning electron microscope image of CXCL10‐encapsulated nanoparticles hitchhiked on the surface of red blood cells. Scale bar, 1 μm. Reproduced with permission.^[^ ^95^ ^]^ Copyright 2021, Springer Nature. (D) Schematic illustration of the combinational therapy with Vadimezan and anti‐PD‐1 antibody‐loaded platelets to treat tumor metastasis. Reproduced with permission.^[^ ^103^ ^]^ Copyright 2021, The Author(s)

Targeting tumor‐associated fibroblasts to improve CAR‐T cell therapy has emerged with the potential to regulate abnormal ECM and facilitate effector T cell infiltration. Fibroblast activation protein (FAP), as one of the overexpressed transmembrane serine proteases in tumor‐associated fibroblasts, becomes a good candidate for tumor‐associated fibroblast targeting due to its great selectivity.^[^ ^89^ ^]^ FAP CAR‐T cells showed the FAP‐specific cytotoxicity against FAP+ stromal cells and an effective tumor growth inhibition ability as a single treatment.^[^ ^90^ ^]^ Also, Kakarla et al. showed the improved efficacy of the combination treatment of FAP CAR‐T cells and EphA2 CAR‐T cells, leading to the clearance of FAP+ tumor‐associated fibroblasts and enhancing the treatment outcome of CAR‐T cells targeting the tumor‐associated antigen EphA2 locally and systemically.^[^ ^91^ ^]^ Matrix components such as collagens and HA are also being investigated as possible targets to overcome physical barriers and further facilitate cancer immunotherapy using cell‐based delivery systems. For example, collagenases and matrix metalloproteinases (MMPs) are two main enzymes that could degrade collagens and remodel the stiff ECM.^[^ ^92^, ^93^ ^]^ Zhang et al. creatively designed a two‐region chimeric antigen receptor for macrophages as the adoptive macrophage therapy to target tumor tissues and destroy natural ECM physical barriers (Figure 2A,B).^[^ ^94^ ^]^ The extracellular region of the chimeric antigen receptor was able to target breast cancer biomarker human epidermal growth factor receptor 2 (HER2), endowing macrophages with tumor targeting ability. And CD147, as the ECM metalloproteinase inducer, was designed as the intracellular region of the chimeric antigen receptor to upregulate synthesis and expression of cellular MMPs. After the injection of modified macrophages in the HER2 4T1 mouse model, the results indicated that this adoptive macrophage treatment assisted by MMP‐mediated collagen degradation substantially reduced the tumor progression, as evidenced by the average weight of tumors from control macrophage was approximately twice as large as that from CAR‐147 macrophage‐treated mice. Besides the direct modulation of tumor‐associated fibroblasts and ECM components, another promising method for locally recruiting effector immune cells and increasing immune cell penetration into tumor parenchyma is the site‐specific accumulation of chemokines like CXCL10 at tumor regions (Figure 2C).^[^ ^95^ ^]^ The CXCL10‐encapsulated nanoparticles hitchhiked on the surface of red blood cells were released selectively at the metastatic lung tissue, resulting in chemokine gradients that enhanced immune cell infiltration locally. Similarly, Nishio et al. developed CCL5 and IL‐15 loaded oncolytic adenovirus that could infect tumor cells to ectopically express CCL5.^[^ ^96^ ^]^ Infected tumor cells that expressed CCL5 enhanced the effective migration of infused CAR‐T cells, which increased anti‐tumor activity.

Another physical obstacle to immunotherapy is the abnormal vasculature at tumor sites, which supplies nourishment to tumor cells facilitating tumor development and metastasis while simultaneously preventing immune cell infiltration.^[^ ^97^ ^]^ Pro‐angiogenic factors, for example, vascular endothelial growth factor (VEGF) and epidermal growth factor‐like protein 7, restrict leukocyte‐endothelial interactions that are essential for immune effector cell penetration into the tumor parenchyma, and also hinder the presentation of tumor‐associated antigens by suppressing dendritic cell maturation, given the immunosuppressive environment generated by abnormal tumor vascular networks.^[^ ^59^, ^98^ ^]^ Hypoxia due to inadequate oxygen delivery caused by aberrant vascularization has also been shown to recruit immunosuppressive cells in the tumor microenvironment, including tumor‐associated macrophages (TAMs), regulatory T cells (Tregs), and myeloid‐derived suppressor cells (MDSCs).^[^ ^99^ ^]^ Thus, given the importance of tumor vascularization in the generation and function of anti‐cancer immune response, combining anti‐angiogenic treatment with cancer immunotherapeutics has emerged as a new trend for controlling tumor growth and improving patient survival. Especially, enhancing therapeutic efficiency by normalizing tumor blood vessels to increase the quantity of infiltrating T cells demonstrated a remarkable anti‐tumor performance in immune cell‐based drug delivery systems for cancer immunotherapy.^[^ ^100^, ^101^ ^]^ For example, Chinnasamy et al. reported that T cell combinational treatment using two distinct targeting antibodies, one targeting VEGFR‐2 on tumor vascular endothelial cells and the other targeting tumor‐associated antigens, such as gp100 or TRP‐1, had a synergistic impact that anti‐VEGF antibodies enhanced the infiltration of tumor antigen‐reactive T cells into melanoma tumor parenchyma, extending the survival time of tumor‐bearing mice significantly.^[^ ^102^ ^]^ The results showed that when mice were co‐administered with anti‐VEGFR2 CAR–T cells, the absolute number of Thy1.1 + Tg‐Pmel T cells was 25.3‐ and 12.7‐fold higher in the spleen and tumor tissues on day 3, respectively. In addition to the immune cell‐based delivery strategies, Li et al. developed the combination treatment of an anti‐angiogenesis treatment with Vadimezan and anti‐PD‐1‐loaded platelets, taking advantage of hemorrhage‐triggered platelet recruitment, to fight against tumor metastasis by unleashing T cell‐based anti‐tumor immune response (Figure 2D).^[^ ^103^ ^]^ Vadimezan‐based therapy damaged the tumor vasculature locally, triggering a coagulation cascade that attracted anti‐PD‐1 antibody‐carried platelets. The engineered platelets accumulated at tumor sites could be activated and released anti‐PD‐1 antibodies locally in the form of platelet‐derived microparticles, invigorating T cell activities against tumor cells. In general, overcoming physical obstacles to cancer immunotherapy with cell‐based delivery techniques utilizing various types of cells as delivery vehicles has shown promising therapeutic results, particularly when treating solid tumors with aberrant tumor vascularization and ECM dysregulation.

### 3.2 Tumor immunosuppressive microenvironment with immunosuppressive cells and cytokines

Two major characteristics that govern the tumor immunosuppressive microenvironment, which results in a chronic inflammatory state and anti‐tumor immunosuppression, are immunosuppressor cells and immunosuppressive cytokines. Immunosuppressive cells, including MDSCs, Tregs, and M2 polarized macrophages, promote tumor progression and metastasis by creating a pre‐metastatic anti‐inflammatory microenvironment, enabling epithelial‐mesenchymal transition and promoting tumor angiogenesis.^[^ ^18^ ^]^ Meanwhile, the tumor‐induced cytokines secreted by immunosuppressive cells and other immune‐related cells in the tumor microenvironment would also modulate the anti‐tumor immunity and facilitate the tumor growth.^[^ ^104^ ^]^ Studies have demonstrated that transforming growth factor‐β (TGF‐β),^[^ ^105^ ^]^ interleukin (IL)‐4,^[^ ^106^ ^]^ IL‐8,^[^ ^107^ ^]^ and IL‐10^[^ ^108^ ^]^ are crucial for tumor development and immunological regulation. Hence, treatment strategies targeting immunosuppressor cells and immunosuppressive cytokines are thought to be potential approaches for boosting anti‐tumor immunity induced by cancer immunotherapies, which also affect the bioactivity and function of cell‐based drug carriers.

**FIGURE 3:**
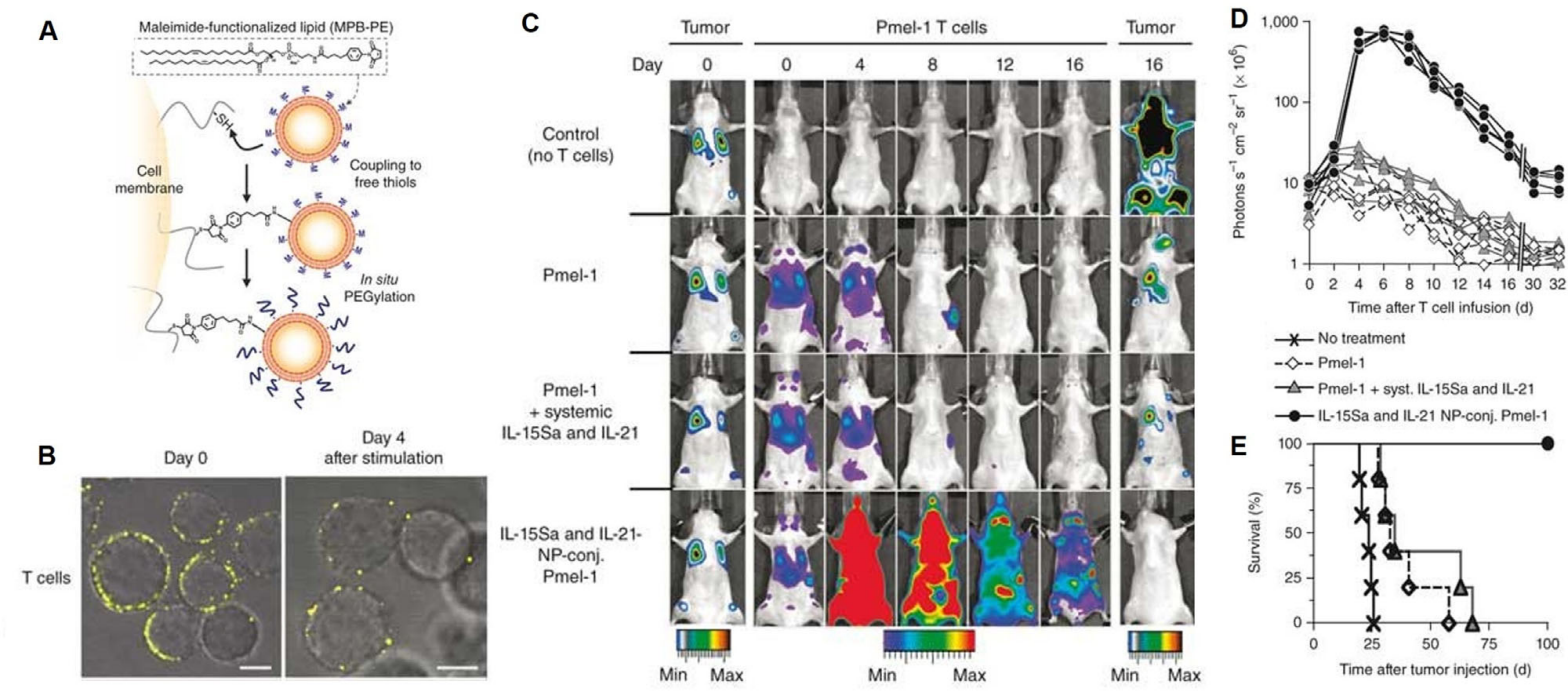
Therapeutic effector T cells engineered with surface‐conjugated cytokine nanoparticles. (A) Schematic illustration of the conjugation of nanoparticles to the surface of T cells. (B) Confocal microscope images of CD8+ T cells conjugated with fluorescence‐labeled nanoparticles at days 0 and 4 after stimulation. Scale bars, 2 μm. (C) In vivo bioluminescence imaging of extG‐luc‐expressing B16F10 tumors and CBR‐luc‐expressing Pmel‐1 T cells. (D) Bioluminescence signal intensities of CBR‐luc T cell were quantified every 2 days after T cell transfer. (E) Survival curve of mice after T cell therapy. Reproduced with permission.^[^ ^38^ ^]^ Copyright 2010, Nature Publishing Group

**FIGURE 4:**
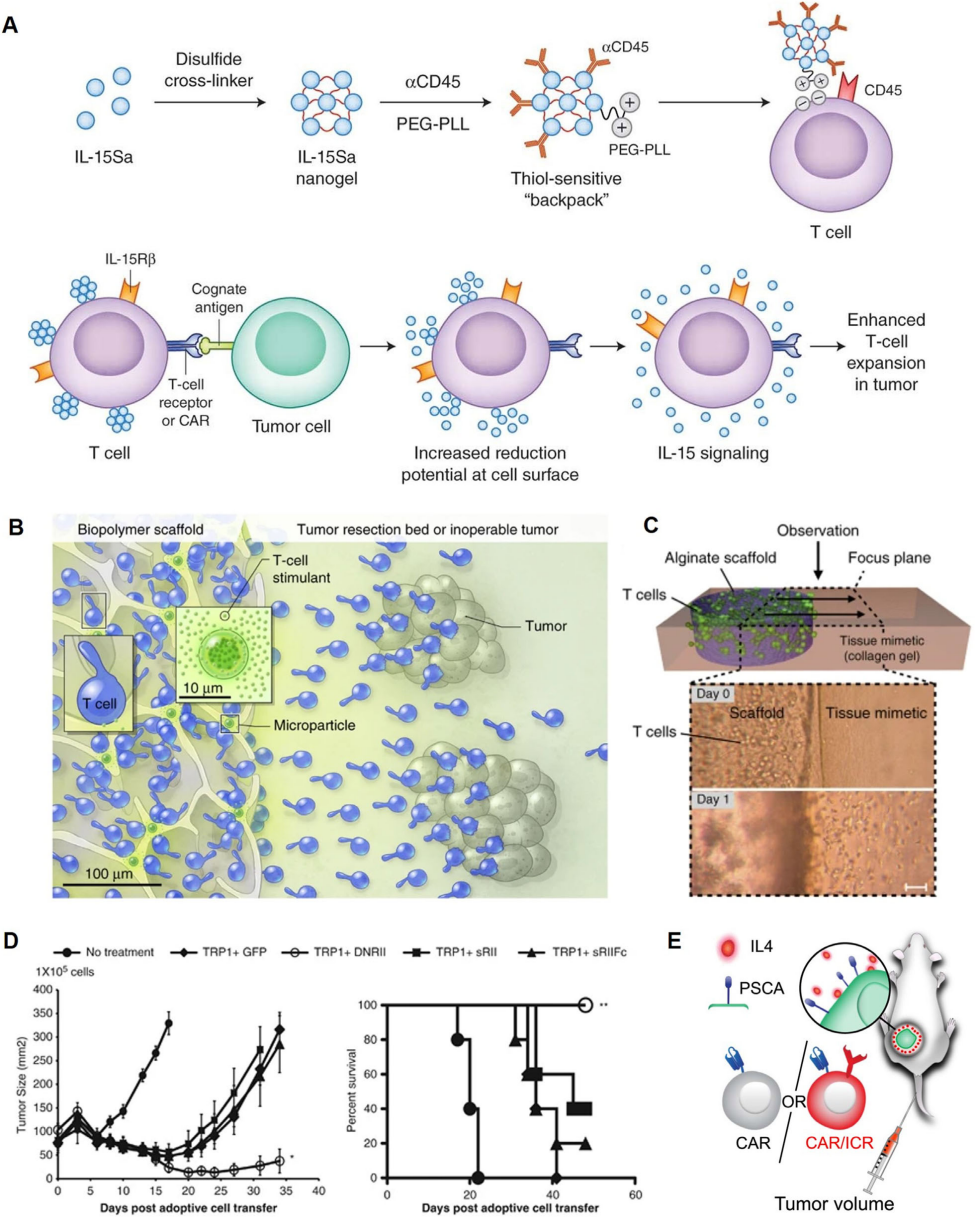
Modulating immunosuppressive cells and cytokines in the tumor immunosuppressive microenvironment. (A) Schematic illustration of the cytokine‐loaded nanogel hitchhiking on the surface of engineered T cells by thiol‐sensitive linkers and T cell modulation mechanisms of IL‐15 cytokines in vivo. Reproduced with permission.^[^ ^114^ ^]^ Copyright 2018, Nature Publishing Group. (B) Schematic illustration of the bioactive polymer carrier loaded with T cells and stimulatory microspheres to facilitate T cell growth and activation at tumor sites. (C) Schematic illustration and corresponding micrographs of the in vitro cell migration from scaffold into the tissue mimetic matrix. Reproduced with permission.^[^ ^115^ ^]^ Copyright 2012, Macmillan Publishers Limited. (D) CD4 T cells co‐expressing tyrosinase‐related protein‐1 and dominant‐negative TGF‐β receptor type II showed enhanced tumor treatment efficacy in B16 melanoma tumor model. Reproduced with permission.^[^ ^117^ ^]^ Copyright 2014, Macmillan Publishers Limited. (E) Schematic illustration of experimental setup that animals engrafted with IL‐4‐producing CAPAN1 cells were treated intravenously with CAR‐T cells engineered with inverted cytokine receptors. Reproduced with permission.^[^ ^118^ ^]^ Copyright 2016, The American Society of Gene and Cell Therapy

Approaches that target various immunosuppressive cells or immunosuppressive cytokine pathways to allow immunity improvement by cancer immunotherapy using cell‐based delivery systems have been developed because of a better knowledge of the critical functions of immunosuppressive cells and related cytokines in anti‐tumor immune modulation. MDSCs accumulating at tumor sites have been proved to hinder the immune activity of T cells, B cells, and natural killer cells, in the meantime promoting the immune escape of tumor cells.^[^ ^109^ ^]^ As a result, either increasing MDSC maturation into dendritic cells or decreasing MDSC accumulation might improve immunotherapeutic efficacy. All‐trans retinoic acid (ATRA) could enhance tumor MDSC development into mature DCs while decreasing the suppressive ability of granulocytic MDSCs. Therefore, Long et al. utilized this characteristic to improve CAR‐T cell therapy engineered with CD19‐specific chimeric antigen receptors with the aid of ATRA.^[^ ^110^ ^]^ Sunitinib, a receptor tyrosine kinase inhibitor, was also used to improve the anti‐tumor efficacy of autologous DCs‐based immunotherapy, showing excellent tolerability and supportive immunologic responses as well as an extension of median and long‐term survival of tumor‐bearing mice.^[^ ^111^ ^]^ In the case of other immunosuppressor cells, such as Tregs, which primarily suppress effector T cells, cytokines including IL‐7, IL‐15, and IL‐21 have been used as regulators to modulate activities of Tregs.^[^ ^112^, ^113^ ^]^ The concurrent delivery of adjuvant cytokines with engineered cells could be achieved by stably attaching cytokine‐encapsulated nanoparticles to cell surfaces via surface thiol groups (Figure 3).^[^ ^38^ ^]^ In order to enhance cooperative in vivo T cell expansion, IL‐15 and IL‐21 were co‐loaded into multilamellar lipid nanoparticles, and melanoma‐specific carrier T cells exhibited excellent tumor elimination capacity against B16F10 melanoma. Besides cytokine nanoparticles, cytokines were also able to be designed as nanogel backpacks by Irvine group, which could hitchhike on the membrane of engineered T cells by reduction‐sensitive chemical linkers (Figure 4A).^[^ ^114^ ^]^ Once T cells reached the tumor site, the release of cytokine from nanogels was induced by T cell activation‐triggered reduction potential on the cell surface, which interacted with disulfide bonds in nanogels, displaying the T cell modulation and high anti‐tumor effectiveness. Besides, with the assistance of soluble IL‐15 superagonists incorporated into physical voids of scaffolds, Stephan et al. created a bioactive polymer carrier capable of transporting and growing tumor‐reactive T cells to significantly increase anti‐cancer ability of immune cells (Figure 4B,C).^[^ ^115^ ^]^ Notably, adoptive cell transfer of ex vivo expanded Tregs has shown tremendous promise in suppressing autoimmune disease and prolonging murine allograft life with the help of IL‐2 nanoparticles, despite the fact that Tregs are not ideal for anti‐cancer immunotherapy.^[^ ^116^ ^]^ Furthermore, besides therapies that are targeting immunosuppressive cells, immune‐inhibitory cytokines like TGF‐β (Figure 4D) and IL‐4 (Figure 4E) have also proven to be great targets for enhancing therapeutic outcomes of adoptive cell therapy.^[^ ^117^, ^118^ ^]^ Engineering with inverted cytokine receptors, which could invert the inhibitory effect of cytokines and signaling inhibition substantially enhancing tumor treatment effectiveness, enabled CAR‐T cell treatments against a variety of malignancies. Overall, enhancing cancer immunotherapy with cell‐based delivery strategies by modulating the immunosuppressive microenvironment composed of immunosuppressive cells and cytokines has recently emerged as an appealing approach, though the complicated and paradoxical immunological roles of immunosuppressive cells and cytokines need to be further investigated for future clinical applications.

### 3.3 Upregulation of immunosuppressive signals

Immune evasion of tumor cells under T cell surveillance owing to the overexpression of immunosuppressive ligands on tumor cell surfaces severely inhibits anti‐tumor immunotherapy efficacy. T cell‐based adaptive immunity is critical for establishing an effective and long‐term immune response that protects human bodies against tumor cell evasion, allowing cancer immunotherapy to reach its maximum therapeutic potential.^[^ ^119^ ^]^ However, the existence of immune checkpoint pathways negatively regulates effector T cells, causing T‐cell anergy so that anti‐tumor immune response is durably limited at tumor sites.^[^ ^16^ ^]^ As a result, using immune checkpoint inhibition as an adjuvant treatment to target inhibitory checkpoint receptors to unleash the suppressed anti‐tumor activity of cancer immunotherapies shows promises in raising the response rate of immunotherapeutics.^[^ ^120^ ^]^ Several immune checkpoint inhibitors, such as anti‐PD‐1/PD‐L1, CTLA‐4, anti‐lymphocyte activation gene 3, and anti‐T cell immunoglobulin and mucin‐3 antibodies, have been extensively studied and are currently being tested in late‐stage clinical trials to treat a variety of cancers.^[^ ^121^ ^]^ Because of flexible modification methods and unique biofunctionalities of cell carriers, combining immune checkpoint inhibitors with cell‐based delivery systems using various techniques such as genetically cell engineering and cell surface decoration is recognized as an effective strategy to facilitate cancer immunotherapeutics.

**FIGURE 5:**
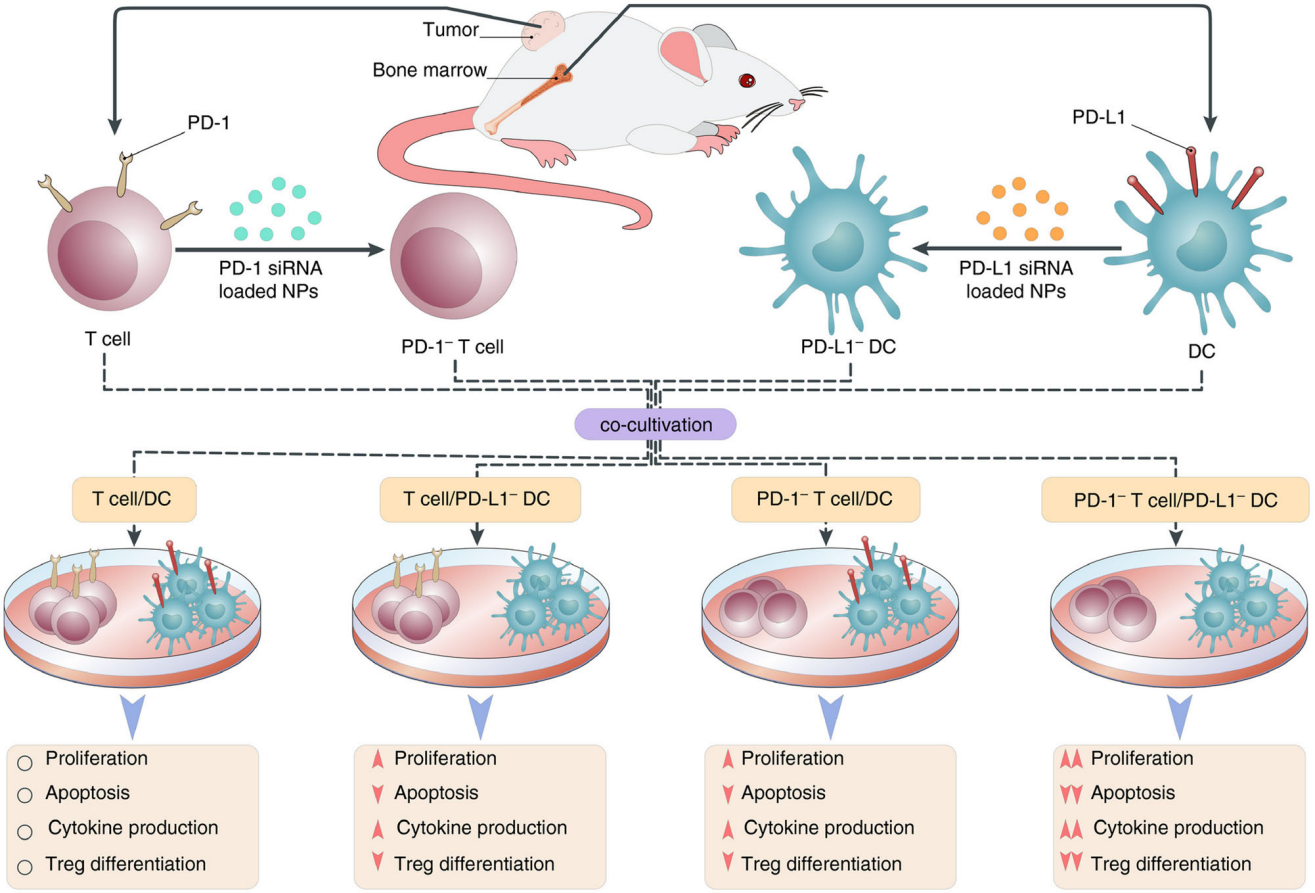
Schematic illustration of PD‐1/PD‐L1 pathway inhibition‐enhanced T cell responses to dendritic cells, promoting response rate and survival. Reproduced with permission.^[^ ^128^ ^]^ Copyright 2019, John Wiley & Sons Ltd

**FIGURE 6:**
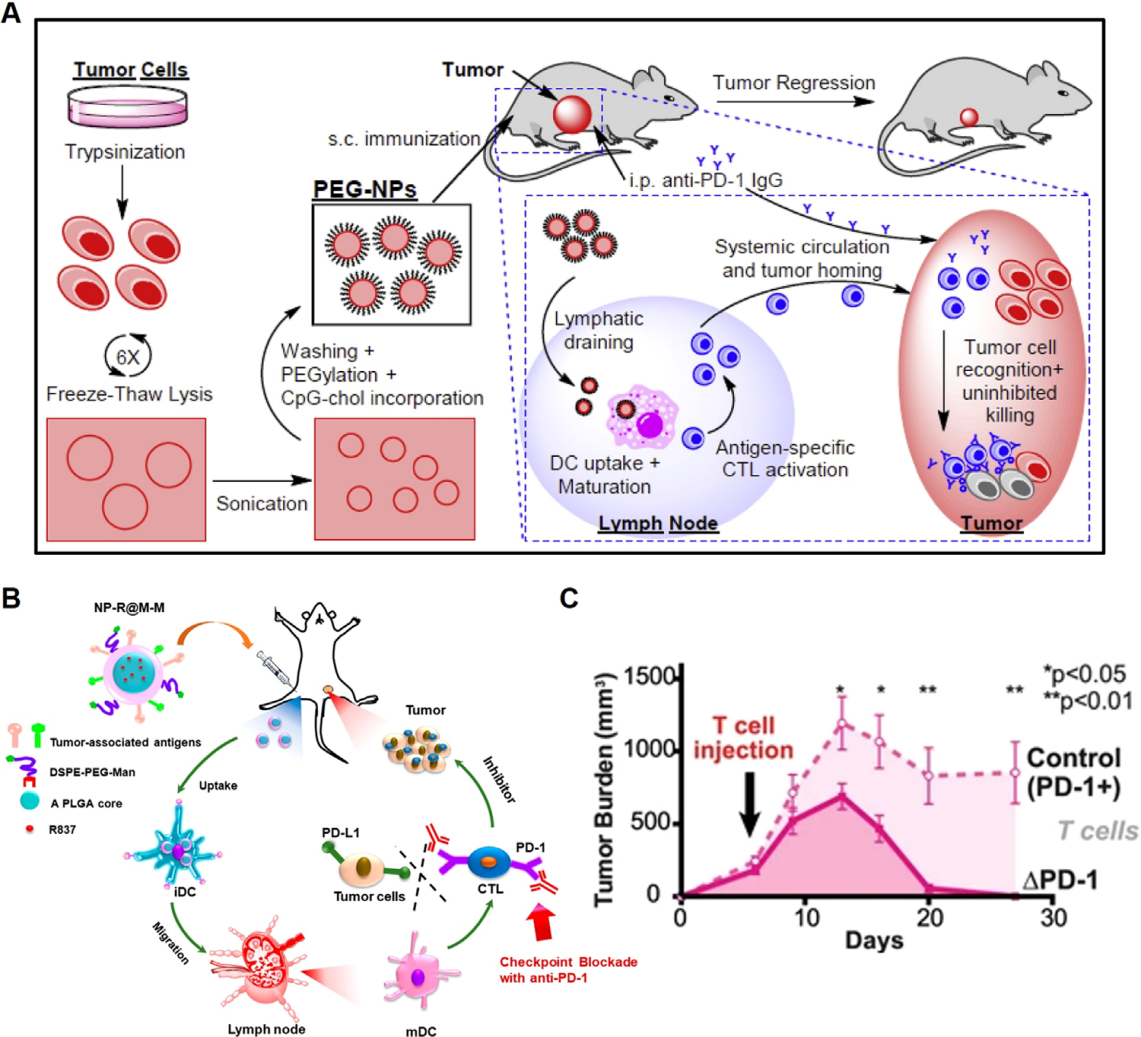
Overcoming the upregulation of immunosuppressive pathways. (A) Schematic illustration of the preparation of polyethylene glycol‐coated cancer cell membrane‐based nanoparticles and the combinational therapeutic strategy of immune checkpoint blockade and nanoparticles. Reproduced with permission.^[^ ^129^ ^]^ Copyright 2018, Elsevier Ltd. (B) Schematic illustration of the structure of tumor cell membrane‐coated nanoparticles loaded with toll‐like receptor 7 agonists and how these treatment systems regulate anti‐tumor immunity in vivo. Reproduced with permission.^[^ ^130^ ^]^ Copyright 2018, American Chemical Society. (C) Anti‐CD19 CAR‐T cells with PD‐1 deficiency had great anti‐tumor activity against subcutaneous CD19+ PD‐L1+ tumor xenografts. Reproduced with permission.^[^ ^132^ ^]^ Copyright 2017, The Author(s)

**FIGURE 7:**
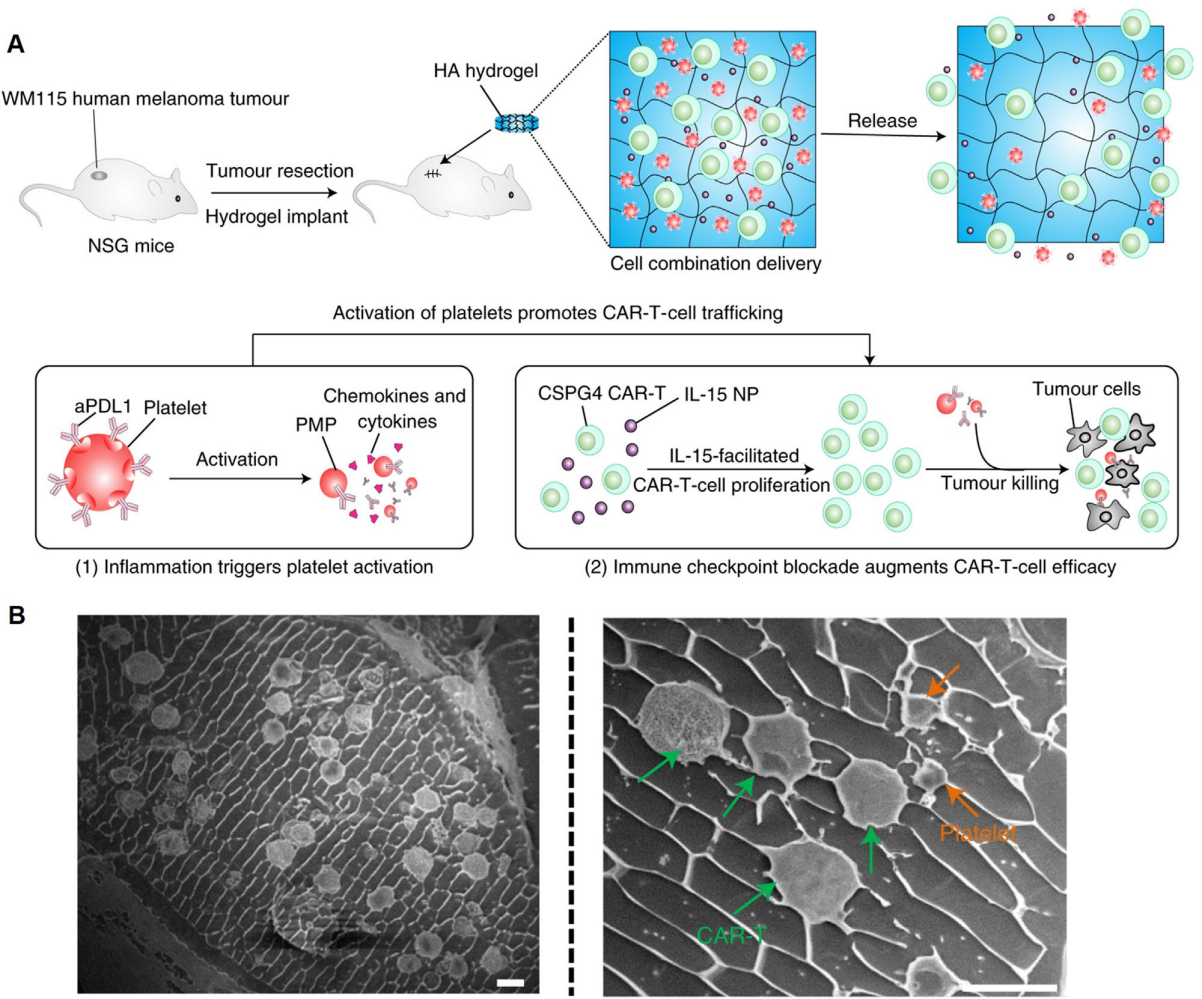
(A) Schematic illustration of the biodegradable HA hydrogel implantation and the mechanisms of platelet activation‐triggered aPD‐1 release and promoted CAR‐T cell activity. (B) Cryo‐scanning electron microscopy imaging of aPD‐1‐conjugated platelets and CAR‐T cells co‐loaded hydrogel. Scale bars, 10 μm. Reproduced with permission.^[^ ^133^ ^]^ Copyright 2021, The Author(s)

Therapeutic cancer vaccines, a type of cancer immunotherapy, utilize tumor‐specific antigens, DNA, or mRNA to develop an efficient anti‐tumor immunity against specific types of tumor cells, relying on T cell‐mediated adaptive immunity in particular.^[^ ^122^ ^]^ The immune response elicited by therapeutic cancer vaccines is dependent on two components: one is the supply of tumor‐associated antigens, and the other is antigen‐presenting cell, which activates the adaptive immune response by presenting antigen to T cells. Therefore, cell‐based delivery strategies are frequently applied in the development of therapeutic cancer vaccines, especially cancer cell membrane‐coated nanoparticles as tumor antigen resource,^[^ ^123^ ^]^ and dendritic cell vaccination since dendritic cells are the most effective antigen presenting cells in the immune system.^[^ ^124^ ^]^ However, the response rate of dendritic cell vaccination itself is only lower than 15%, and cancer cell membrane‐coated nanoparticles could not have the most effective immune response under a strong immunosuppressive environment.^[^ ^125^, ^126^ ^]^ As a result, the concept of utilizing immune checkpoint inhibition to improve the therapeutic effectiveness of cancer vaccines has gained popularity. For example, Teng et al. combined a hepatocellular carcinoma‐specific dendritic cell vaccine and anti‐PD‐L1 antibodies and achieved enhanced anti‐tumor immune responses.^[^ ^127^ ^]^ Hassannia et al. further confirmed the underlying mechanisms of how PD1/PD‐L1 pathway silencing was related to the treatment efficacy of dendritic cell vaccine, showing that PD‐L1 inhibition could upregulate the expression of HLA‐DR and CD86 to prime T cells and could also effectively enhance the migration of dendritic cells toward CCL21 chemokine (Figure 5).^[^ ^128^ ^]^ As for cancer cell membrane‐based vaccine delivery approaches, when tumor‐bearing mice were given a combination of cancer cell membrane‐coated nanoparticles and anti‐PD‐1 antibody immunotherapy, mice survived better than individuals given solely cancer cell membrane‐based vaccines (Figure 6A).^[^ ^129^ ^]^ Moreover, cancer cell membrane‐based vaccines additionally encapsulating various immune‐adjuvant nanoparticles like toll‐like receptor 7 agonist^[^ ^130^ ^]^ and CpG oligodeoxynucleotide 1826^[^ ^123^ ^]^ were also demonstrated to have better treatment outcomes when integrating with immune checkpoint inhibitors (Figure 6B). Aside from cancer vaccines, several studies have emphasized the synergistic impact of adoptive cell transfer and immune checkpoint blockade by genetically engineering CAR‐T cells with immune checkpoint inhibitors as well.^[^ ^131^, ^132^ ^]^ Rupp et al. reported that PD‐1 deficient anti‐CD19 CAR‐T cell‐mediated killing of tumor cells in vitro was increased, as was the elimination of PD‐L1‐expressing tumor xenografts in vivo, which proved that the PD‐1 disruption could enhance anti‐tumor efficacy of CAR‐T cell therapy (Figure 6C).^[^ ^132^ ^]^ Hu et al. also reported that a biodegradable hydrogel could be used as a reservoir for sustainedly releasing melanoma‐targeting CAR‐T cells, IL‐15‐loaded nanoparticles, and human platelets coupled with anti‐PD‐L1 antibodies when placed into the tumor‐resection cavity in a melanoma tumor‐bearing animal model (Figure 7).^[^ ^133^ ^]^ The adjuvant IL‐15, which was released continuously from nanoparticles, and aPD‐L1 antibodies, which were delivered by platelet‐derived microparticles, worked together to enhance CAR‐T cell viability and protect CAR‐T cells from exhaustion, allowing them to retain anti‐tumor immune activities. The quantitative bioluminescence intensity of tumors treated with the combinational treatment was more than 60 times lower than other treatment groups at week 3. Promising therapeutical outcomes were observed, with local tumor recurrence being significantly reduced and abscopal anti‐tumor effects being promoted. Furthermore, helper‐dependent adenoviruses that express PD‐L1 inhibitors could improve the anti‐tumor impact of oncolytic immunotherapy utilizing engineered adenovirus‐loaded mesenchymal stromal cells, resulting in the reduced tumor progression in an orthotopic lung cancer mouse models.^[^ ^134^ ^]^ To summarize, immune checkpoint blockade is effective for facilitating anti‐tumor efficacy of different cancer immunotherapy using cell‐based delivery techniques to overcome overexpression of immunosuppressive ligands.

### 3.4 Metabolic restriction

Due to the continual growth of tumor tissues, unique regional metabolic patterns in the tumor microenvironment play a key role in immunomodulation and inflict metabolic stress on immune effector cells, altering their anti‐tumor immune behaviors. The rapid proliferation and division of tumor cells demand a level of oxygen and glucose owing to a high rate of glycolysis, which results in the hypoxia and lactate accumulation in tumor regions.^[^ ^21^ ^]^ The low oxygen level in the tumor microenvironment limits T cell growth and anti‐tumor effects and facilitates cancer metastasis and immune resistance.^[^ ^135^ ^]^ In addition to the inhibitory effect of hypoxia on effector T cells, hypoxia and low glucose levels at tumor sites can attract immunosuppressive cells, such as MDSCs and Tregs. Due to the recruitment of MDSCs and Tregs, indoleamine 2,3‐dioxygenase (IDO), the immunoregulator related to tryptophan metabolism, is elevated at tumor sites, which develops anti‐inflammatory responses and immunological tolerances to apoptotic cells.^[^ ^136^ ^]^ Meanwhile, during tumor metabolism, ROS are produced, causing oxidative stress and disrupting adoptive cell therapy's anti‐tumor efficacy.^[^ ^137^ ^]^ Given the importance of metabolism profile and hypoxia in the tumor microenvironment, shielding cell‐delivered therapeutics from environmental stress or using environmental stress to inhibit tumor cell proliferation might be a viable strategy for improving immunotherapy outcomes.

**FIGURE 8:**
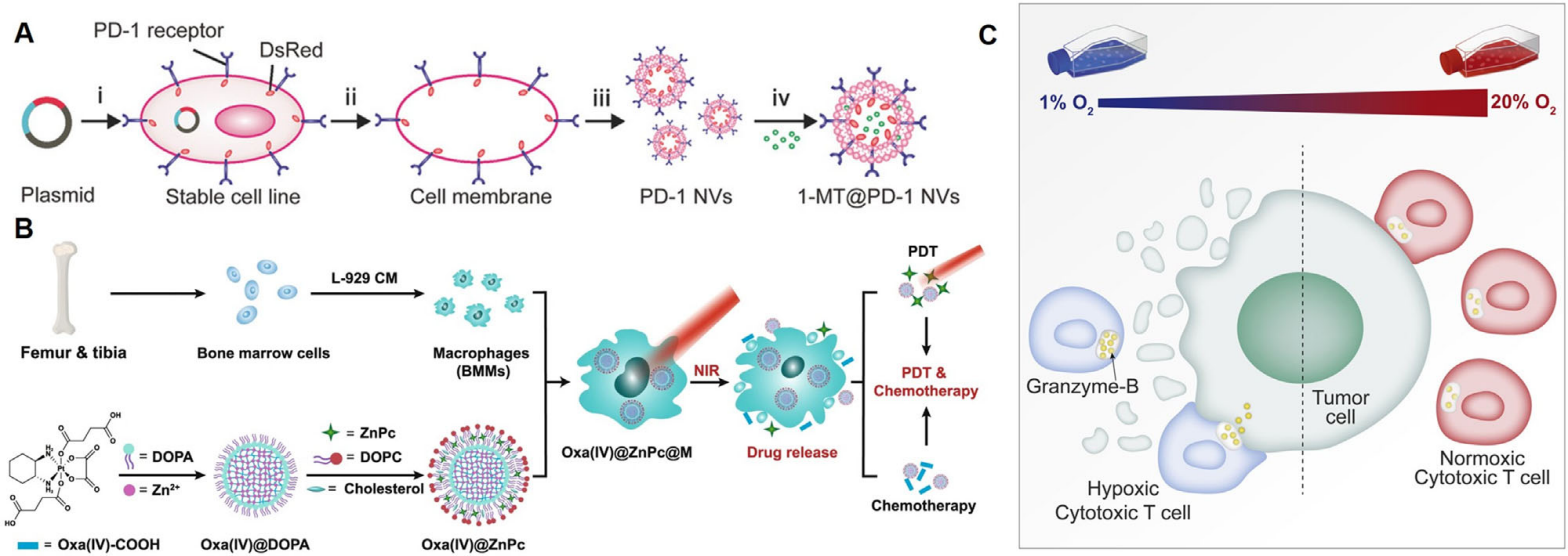
Overcoming metabolic restrictions. (A) Schematic illustration of the preparation process of 1‐MT‐loaded nanovesicles engineered with PD‐1 receptors. Reproduced with permission.^[^ ^139^ ^]^ Copyright 2019, WILEY‐VCH Verlag GmbH & Co. KGaA, Weinheim. (B) Schematic illustration of the preparation process of engineered macrophages carrying oxaliplatin prodrugs and photosensitizers. Reproduced with permission.^[^ ^142^ ^]^ Copyright 2021, The Author(s). (C) Schematic illustration of cytotoxic T cells activated under hypoxic conditions with improved anti‐tumor cytotoxicity. Reproduced with permission.^[^ ^144^ ^]^ Copyright 2017, Elsevier Inc.

Targeting immunosuppressive IDO expressed by tumor cells and dendritic cells has shown great T cell‐based immune enhancement ability when combined with other treatment modalities, such as radiotherapy, chemotherapy, and immune checkpoint blockade.^[^ ^138^ ^]^ Therefore, Zhang et al. developed PD‐1‐presenting nanovesicles derived from 293T cell membranes encapsulating small‐molecule IDO inhibitors called 1‐methyl‐tryptophan (1‐MT) (Figure 8A).^[^ ^139^ ^]^ The cumulative effects of 1‐MT‐mediated IDO inhibition and PD‐1/PD‐L1 pathway blockage synergistically increased CD8+ T cell infiltration and a considerably improved T cell‐based immune response against melanoma. Aside from IDO overexpression, increased ROS levels at tumor sites are another impediment to anti‐tumor immune activity, particularly CAR‐T cell therapy. To retain their anti‐tumor potential, T cells co‐expressing catalase along with a CAR were designed to overcome high levels of ambient ROS with higher amounts of intracellular catalase, compared with normal CAR‐T cells.^[^ ^140^ ^]^ However, because it is difficult for cell‐based immunotherapies to avoid damage caused by high ROS levels after delivery, researchers ingeniously exploited ROS‐induced damage to kill tumor cells using cell‐based delivery methods to produce excessive ROS artificially.^[^ ^141^, ^142^, ^143^ ^]^ For example, Huang et al. reported that engineered M1 macrophages carrying oxaliplatin prodrug and photosensitizer were able to be activated by near‐infrared light to generate cytotoxic ROS (Figure 8B).^[^ ^142^ ^]^ Photodynamic treatment was able to eliminate tumor cells, which then produced tumor‐associated antigens, triggering an anti‐tumor immune response and facilitating anti‐PD‐L1 immune checkpoint blockade. In both 4T1 and EMT6 mice models, primary and bone metastatic cancers were eliminated. Engineered macrophages with oxaliplatin prodrug and photosensitizer‐treated mice had the greatest frequency of CD8 + T cells infiltration, which was 5.9‐ and 10.4‐fold greater than the PBS group, in the 4T1 and EMT6 model respectively. Besides, the overall survival rate of tumor‐bearing mice was improved with minimal systemic toxicity. Furthermore, training T cells to acclimate to hypoxia at tumor sites can be another helpful strategy for increasing the anti‐tumor efficacy of effector immune cells. Gropper et al. activated cytotoxic T lymphocytes under hypoxic conditions to generate hypoxic cytotoxic T cells (Figure 8C).^[^ ^144^ ^]^ Results demonstrated that mice treated with hypoxic cytotoxic T cells showed considerable tumor regression and a longer survival time than mice treated with normal cytotoxic T cells. To summarize, since a lot of immunotherapies rely on metabolic control to be effective, a better knowledge of how immunometabolism influences therapeutic outcomes is required to create novel treatment techniques that make cell‐based delivery systems more feasible.

## 4 CONCLUSION

Nowadays, due to a better understanding of biological and structural specialties of various cells, including immune cells, blood cells, and stem cells, the potential delivery applications of cell‐based delivery systems in cancer immunotherapy have been revealed and widely implemented, with great success in improving delivery efficacy and optimizing therapeutic outcomes of immunotherapeutic drugs. Clinical investigation of cancer immunotherapeutics transported by flexibly engineered cell carriers displays exciting advantages over traditional drug delivery systems, showing board utilization in various cancers ranging from blood cancer to solid tumors.^[^ ^145^, ^146^ ^]^ Compared with traditional delivery systems, engineered cell carriers are more biocompatible, and can offer a variety of benefits such as low immunogenicity, evading immune clearance, site‐specific accumulation, inherent interaction with tumor cells, and immune modulation activities.^[^ ^40^ ^]^ Moreover, various engineering methods endow cell‐based carriers with the potential to integrate the functionalities of previously developed systems, which bring new opportunities for addressing unmet challenges in the clinic.^[^ ^114^, ^115^ ^]^ Cell‐based carriers pave a way for precision and personalized medicine by engineering patients’ own cells as therapeutics or delivery systems. Furthermore, the recent approval of CAR‐T cells for clinical treatment boosts the development of cell‐based therapy, which will ignite a promising research frontier for engineering cells for effective and precision treatment.^[^ ^147^ ^]^

However, there is still a long way to go to make full use of cell‐based delivery carriers in clinical applications. One factor limiting the accessibility of cell‐based carriers is the requirement of skilled professionals and high‐standard operating conditions for cell engineering. For personalized cell‐based formulations that are customized for each individual, sophisticated procedures for engineering cells may lead to the considerable cost of time and money that restrict the expansion of their clinical applications. For “off‐the‐shelf” products, thorough investigations are needed to optimize the conditions for storage and transportation that can preserve the activity and function of cells. In addition, correlation between in vitro activity and in vivo performance of cell‐based therapy should be carefully evaluated to avoid limited therapeutic outcomes as well as potential side effects in the clinical setting. Therefore, unpredictable immunogenicity to cell‐based delivery carriers, varying treatment response rates among patients, and an unknown optimal dosage are still challenges that need to be addressed before cell carrier‐facilitated cancer immunotherapy may be used in clinical trials. Furthermore, anti‐tumor immune resistance mechanisms, such as impaired immune cell infiltration, immunosuppressive cell recruitment, metabolic microenvironment change, and immunosuppressive pathway upregulation, obstruct therapeutic efficacy of cancer immunotherapy. As a result, there has been a considerable interest in employing various strategies to overcome hurdles to effective cancer immunotherapy with cell‐based carriers (Table 2). With the development of advanced manufacture technology, the establishment of quality control standards, and the maturity of the regulatory system, cell‐based carriers are bound to have ample scope for application and will provide an option for cancer immunotherapy in the future.

**TABLE 2:** Summary of the barriers to effective cancer immunotherapy delivered by cell‐based carriers and recent advances in overcoming those barriers

**Barriers to cell‐based delivery of cancer immunotherapy**	**Strategies**
Physical barriers to immune cell infiltration	(i) Targeting the overexpressed FAP in tumor‐associated fibroblasts^[^ ^90^, ^91^ ^]^ (ii) Applying collagenases and MMPs to degrade collagens and remodel the stiff ECM^[^ ^94^ ^]^ (iii) Site‐specific accumulation of chemokines to locally increase immune cell infiltration^[^ ^95^, ^96^ ^]^ (iv) Normalizing tumor blood vasculature^[^ ^102^, ^103^ ^]^
TME with immunosuppressive cells and cytokines	(i) Increasing MDSC maturation into dendritic cells or decreasing MDSC accumulation^[^ ^110^, ^111^ ^]^ (ii) Modulating activities of Tregs with cytokines^[^ ^38^, ^114^, ^115^, ^116^ ^]^ (iii) Engineering cell‐based delivery systems with inverted immunosuppressive cytokine receptors^[^ ^117^, ^118^ ^]^
Upregulation of immunosuppressive signals	Immune checkpoint blockade^[^ ^123^, ^127^, ^128^, ^129^, ^130^, ^131^, ^132^, ^133^, ^134^ ^]^
Metabolic restriction	(i) Targeting immunosuppressive IDO with IDO inhibitors^[^ ^139^ ^]^ (ii) ROS level increasement‐induced tumor cell damage^[^ ^142^ ^]^ (iii) Training T cells to acclimate to hypoxia tumor region^[^ ^144^ ^]^

Notably, with the growing interest and extensive study of combination cancer immunotherapy, using cell‐based carriers as excellent platforms to combine two or more immunotherapeutics has become a new trend, such as immune checkpoint blockade, adoptive cell transfer, and cytokine‐based immunotherapy.^[^ ^148^, ^149^ ^]^ Combining the benefits of several immunotherapeutics, cell‐delivered combinational immunotherapy shows excellent synergetic or combinational anti‐tumor effects, aided by the positive activities of cell carriers. For example, Xie et al. presented an interesting study that employed leukocyte membrane‐coated magnetic nanoclusters to magnetically target tumor sites delivering loaded TGF‐β inhibitors and membrane‐anchored PD‐1 antibodies, taking advantage of membrane camouflage to prolong the circulation time and enhance therapeutic efficacy.^[^ ^150^ ^]^ It cannot be overstated that the successful integration of combination cancer immunotherapy using cell carriers is dependent in large part on significant advances in multidisciplinary fields such as bioengineering, chemistry, and material sciences, which have resulted in more and better cell/cell‐membrane engineering and modification methods. For instance, the conjugation method based on key‐lock interaction between proteins provides new perspectives on cell surface modification.^[^ ^26^, ^151^, ^152^ ^]^ As a result of evolving new technologies, the viability and functionalities of carrier cells might be better preserved, and spatiotemporal release of loaded immunotherapeutics will be achieved.

Furthermore, how to design treatment regimens with the consideration of the heterogeneity of tumor tissue and the individual difference in immune response is essential for improving the anti‐tumor efficacy of precision medicine among patients. The anti‐tumor immune response modulated by treatment modalities may differ, and this may be affected in part by the patient's own metabolic features.^[^ ^21^ ^]^ Studies have shown that diets play an essential role in regulating human body metabolism and gut microbiota with anti‐tumor immunomodulatory effects.^[^ ^153^, ^154^ ^]^ As an adjuvant therapy to improve the treatment result of immunotherapy, manipulating the gut microbiota to modulate systemic anti‐tumor immunity has become a new approach.^[^ ^155^ ^]^ Meanwhile, obesity contributes to anti‐cancer immune regulation through adipokine production and the overexpression of immune checkpoint ligands, which should be taken into account when developing personalized immunotherapy.^[^ ^156^, ^157^ ^]^

Although cell‐based drug delivery systems are promising tools in cancer immunotherapy, showing significant therapeutic efficacy based on the potential of diverse functionalization of cell carriers with biological and chemical methods to overcome the restriction of taking effects, solid preclinical and clinical studies are required for further development with the help of multidisciplinary collaboration. Specifically, a profound understanding of tumor immune‐oncology and versatile technologies in cell engineering will enormously facilitate the translation and application of cell carrier‐based anti‐tumor immunotherapy in the clinical setting.

## CONFLICT OF INTEREST

The authors declare no conflict of interest.
